# Can low-intensity pulsed ultrasound (LIPUS) accelerate bone healing after intramedullary screw fixation for proximal fifth metatarsal stress fractures? A retrospective study

**DOI:** 10.1186/s12891-021-04611-z

**Published:** 2021-08-23

**Authors:** Ryo Murakami, Takaki Sanada, Miyu Inagawa, Hiroki Yoshitomi, Eisaburo Honda, Atsushi Fukai, Hiroshi Iwaso

**Affiliations:** 1 Department of Sports Orthopaedic Surgery, Kanto Rosai Hospital, 1-1 Kizukisumiyoshi-cho, Nakahara-ku, Kawasaki, Kanagawa Prefacture Japan; 2 Faculty of Medical Technology, Teikyo University, Tokyo, Japan

**Keywords:** Low-intensity pulsed ultrasound, LIPUS, Intramedullary screw fixation, Fifth metatarsal, Jones fracture

## Abstract

**Background:**

Intramedullary screw fixation is considered the standard treatment for proximal fifth metatarsal stress fractures. Low-intensity pulsed ultrasound (LIPUS) is a well-known bone-healing enhancement device. However, to the best of our knowledge, no clinical study has focused on the effect of LIPUS for postoperative bone union in proximal fifth metatarsal stress fractures. This study aimed to investigate the effect of LIPUS treatment after intramedullary screw fixation for proximal fifth metatarsal stress fractures.

**Methods:**

Between January 2015 and March 2020, patients who underwent intramedullary screw fixation for proximal fifth metatarsal stress fractures were investigated retrospectively. All patients underwent intramedullary screw fixation using a headless compression screw with autologous bone grafts from the base of the fifth metatarsal. The time to restart running and return to sports, as well as that for radiographic bone union, were compared between groups with or without LIPUS treatment. LIPUS treatment was initiated within 3 weeks of surgery in all cases.

**Results:**

Of the 101 ft analyzed, 57 ft were assigned to the LIPUS treatment group, and 44 ft were assigned to the non-LIPUS treatment group. The mean time to restart running and return to sports was 6.8 and 13.7 weeks in the LIPUS treatment group and was 6.2 and 13.2 weeks in the non-LIPUS treatment group, respectively. There were no significant differences in these parameters between groups. In addition, the mean time to radiographic bone union was not significantly different between the LIPUS treatment group (11.9 weeks) and the non-LIPUS treatment group (12.0 weeks). The rate of postoperative nonunion in the LIPUS treatment group was 0% (0/57), while that in the non-LIPUS treatment group was 4.5% (2/44). However, this difference was not statistically significant.

**Conclusions:**

There were no statistically significant differences regarding the time to start running, return to sports, and radiographic bone union in patients with or without LIPUS treatment after intramedullary screw fixation for proximal fifth metatarsal stress fractures. Therefore, we cannot recommend the routine use of LIPUS to shorten the time to bone union after intramedullary screw fixation for proximal fifth metatarsal stress fractures.

## Background

Proximal fifth metatarsal stress fractures are caused by repetitive loading on the fifth metatarsal bone [1, 2]. The re-union of these fractures is more difficult than that of Jones fractures because of the poor blood supply to the proximal metatarsal diaphyseal region of the fifth metatarsal [2, 3]. Studies following intramedullary screw fixation for these fractures have reported a higher success rate and shortened time to bone union as compared to nonsurgical treatment [4, 5]. Currently, intramedullary screw fixation is considered the standard treatment for these fractures. Moreover, these fractures commonly occur in athletes. Many athletes desire an early return to sports, but the appropriate time for a safe return to sports is still unclear. In stress fractures, return to sports after a bone union is generally advocated [6, 7], and many studies on proximal fifth metatarsal stress fractures have adopted this criterion [8–10]. Early return to sports following intramedullary screw fixation for proximal fifth metatarsal stress fractures does not increase the risk of nonunion, but lengthens the time to bone union [11]. Therefore, an early bone union is important for patients who undergo surgery for these fractures because it leads to a safe and early return to sports and, as a result, meets patient demands.

Low-intensity pulsed ultrasound (LIPUS) is a well-known bone-healing enhancement device. Acoustic pressure waves produced by LIPUS stimulate the bone healing process [12]. Many studies have demonstrated that LIPUS enhances bone healing in fresh fractures, delayed union, and nonunion [13–19]. LIPUS also has a beneficial effect on the repair of stress fractures despite different healing processes between stress fractures and complete fractures [20]. Notably, only a study focusing on LIPUS as a nonsurgical treatment for proximal fifth metatarsal stress fractures have reported good outcome [21]. However, to the best of our knowledge, there are no current studies in the literature that evaluate the effect of LIPUS treatment after surgery for proximal fifth metatarsal stress fractures.

The purpose of this study was to assess whether LIPUS treatment after intramedullary screw fixation for proximal fifth metatarsal stress fractures affects clinical and radiological outcomes. We hypothesized that LIPUS treatment early after intramedullary screw fixation for proximal fifth metatarsal stress fractures accelerates bone healing and leads to an early return to sports.

## Methods

### Patient selection

This study protocol was approved by the institutional review board of Kanto Rosai Hospital (IRB No. 2019–28). Informed consent was obtained from the patients using the opt-out option on our website. A retrospective search was conducted from January 2015 to March 2020 using our institution’s database to identify all patients who underwent intramedullary screw fixation for proximal fifth metatarsal stress fractures (zone 3 fractures according to Lawrence-Bottle classification) [22]. The exclusion criteria were as follows: (1) revision surgery, (2) postoperative wound infection, (3) follow-up in other hospitals, and (4) lost to follow-up until radiographic bone union.

### Surgical procedure and rehabilitation

All patients underwent intramedullary screw fixation using the Acutrak screw (Acumed Inc., Beaverton, Oregon, USA) with autologous bone grafting. An autologous bone graft was harvested from the fifth metatarsal base using an 8G × 10 cm original Jamshidi bone marrow biopsy aspiration needle (Beckton Dickinson, Franklin Lakes, New Jersey, USA) before drilling. An appropriate screw size (4/5 or Plus) that would fit the intramedullary canal was used. Through a small incision over the fracture site, the sclerotic bone was curetted, and the autologous bone graft was crushed and packed into the fracture site.

Postoperatively, patients were allowed to walk via heel gait without a cast. Walking was restarted one week after the surgery, and patients were allowed to run when callus formation was noted on a radiograph. Patients were allowed to return to sports when radiographic bone union was achieved.

### LIPUS treatment protocol

The selection of LIPUS treatment or no treatment was dependent on the patient’s decision after an adequate discussion with the surgeon. Patients who used LIPUS in our study were fitted with the EXOGEN Bone Healing System Express (Bioventus, Durham, North Carolina, USA) and received a maximum of 150 treatments. The device delivers low-intensity pulsed ultrasound in 20-min sessions, which is self-administered by the patient at home once daily. The surgical procedure in this study included bone grafting on the fracture site through a small incision. LIPUS treatment was not started immediately after surgery because the wound corresponded to the fracture site. Therefore, LIPUS treatment was started early after the wound on the fracture site healed and continued until radiographic bone union was achieved in this study. As a result, all patients who used LIPUS started LIPUS treatment within 3 weeks after surgery.

### Evaluation

Medical records were reviewed to confirm eligibility, obtaining baseline demographics (age, sex, height, weight, smoking, Tegner activity scale, time from injury to surgery), Torg’s classification [23], status of being with or without LIPUS treatment, the time needed to start running and return to sports, and further details of the surgery. The primary outcome measures were: time to radiographic bone union, time to start running, and time to return to sports. These items were compared between the groups of patients with and without LIPUS treatment.

Additionally, radiographic callus formation and bone union were evaluated on anteroposterior, oblique, and lateral views using plain radiographs. Radiographic bone union was defined as the disappearance of the fracture line in all views. The definition of return to sports was participation in a match or practice in the form of games in this study.

### Statistical analysis

Statistical analysis was conducted using Bell Curve for Excel (Social Survey Research Information Co., Ltd. Tokyo, Japan). Differences were considered statistically significant at *p* < 0.05. Age, height, weight, Tegner activity scale, time from injury to surgery, screw length, the ratio of the screw length to the fifth metatarsal length, time to start running, time to return to sports, and time to the radiographic bone union between the two groups were compared using the Mann-Whitney U-test. Fisher’s exact test was used to compare sex, smoking, and type of Acutrak screw between the two groups. The chi-squared test was used to compare Torg’s classification between the two groups. A post-hoc sample size calculation was performed based on time to bone union. There was a 0.1-week difference between the two groups in this study, and the standard deviation was 3.29 weeks. Significance level was set at *p* < 0.05. To achieve a power of 80%, this revealed the total sample size needed to detect a difference of 19,340 cases in each group.

## Results

**Fig. 1:**
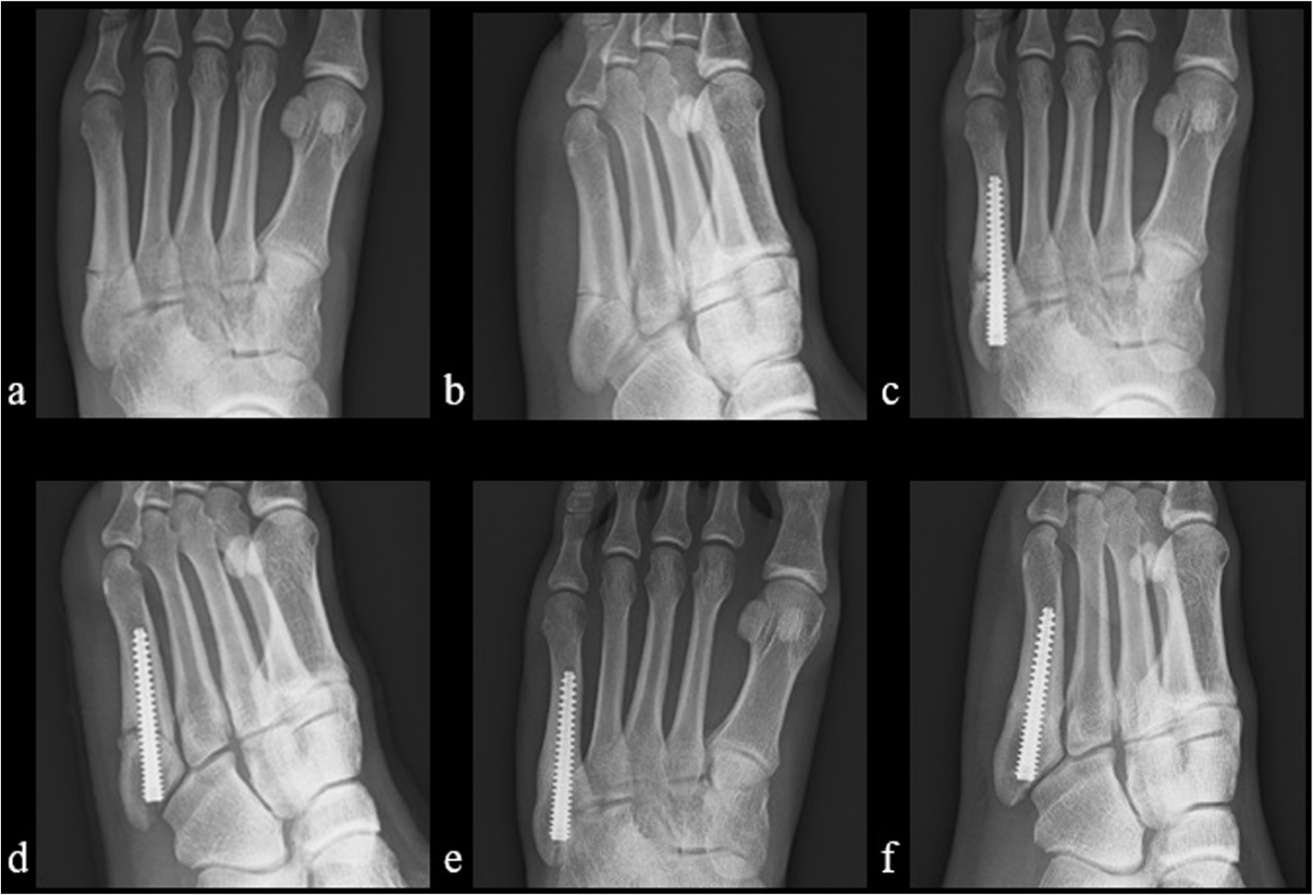
Case presentation. A 17-year-old patient with LIPUS treatment after intramedullary screw fixation. a and b. Radiographs showed proximal metatarsal stress fracture preoperatively (a. anterior-posterior and b. oblique view). c and d. Radiographs in the immediate postoperative period (c. anterior-posterior and d. oblique view). e and f. The radiographs obtained 11 weeks postoperatively showed bone union (e. anterior-posterior and f. oblique view)

**Fig. 2:**
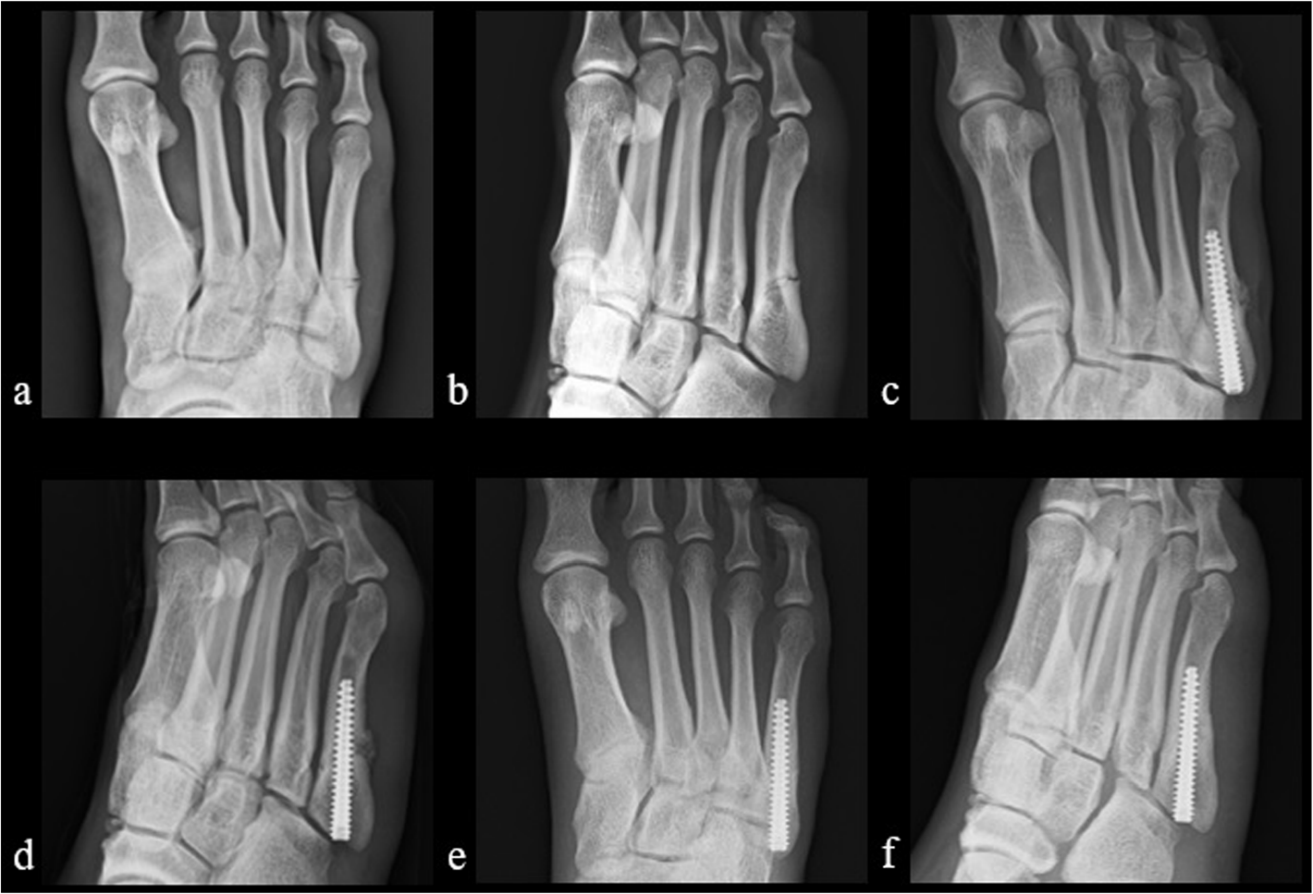
Case presentation. A 19-year-old patient without LIPUS treatment after intramedullary screw fixation. a and b. Radiographs showed proximal metatarsal stress fracture preoperatively (a. anterior-posterior and b. oblique view). c and d. Radiographs in the immediate postoperative period (c. anterior-posterior and d. oblique view). e and f. The radiographs obtained 12 weeks postoperatively showed bone union (e. anterior-posterior and f. oblique view)

One hundred-twenty-two patients (130 ft) who met the inclusion criteria within the study period were investigated retrospectively. Twenty-seven patients (29 ft) were excluded, including three patients (three feet) who had undergone revision surgery, one patient (one foot) who underwent an additional surgery due to postoperative wound infection, 13 patients (13 ft) who were followed up in other hospitals, and 11 patients (12 ft) were lost to follow-up until radiographic bone union. A total of 95 patients (101 ft) were included in this study. A total of 53 patients (57 ft) were assigned to the LIPUS treatment group, and 42 patients (44 ft) were assigned to the non-LIPUS treatment group. There were no significant differences in age, sex, Tegner activity scale, height, weight, smoking, Torg’s classification, and the time from injury to surgery found between the LIPUS treatment group and the non-LIPUS treatment group (Table 1). In addition, there were no significant differences in the type of Acutrak screw, screw length, and the ratio of the screw length to the fifth metatarsal length found between the two groups (Table 2). The mean time to start running and return to sports was 6.8 and 13.7 weeks in the LIPUS treatment group, and that in the non-LIPUS treatment group was 6.2 and 13.2 weeks, respectively. There were no significant differences regarding return to sports and running time parameters between the groups. Moreover, the time to radiographic bone union was not significantly different between the LIPUS treatment group (11.9 ± 3.3 weeks) and the non-LIPUS treatment group (12.0 ± 3.8 weeks), as shown in Figs. 1 and 2. The rate of postoperative nonunion in the LIPUS treatment group was 0% (0/57), while that in the non-LIPUS treatment group was 4.5% (2/44). However, this difference was not noted to be statistically significant. (Table 3).

**Table 1:** Comparison of background characteristics between LIPUS and non-LIPUS groups

		Non-LIPUS treatment group	LIPUS treatment group	*P* value
No. of patients (feet)		42 (44)	53 (57)	
Age at surgery (years)		20.0 ± 5.3	18.9 ± 4.0	0.07
Sex (no. of feet)	Male	42	54	1.00
	Female	2	3	
Height (cm)		174.3 ± 6.0	174.1 ± 5.8	0.97
Weight (kg)		69.7 ± 9.7	69.6 ± 8.0	0.98
Tegner activity scale		8.0 ± 1.0	9.0 ± 0.6	0.38
Smoking (no. of feet)	+	0	1	1.00
	–	44	56	
Torg’s classification (no. of feet)	Type 1	9	10	0.66
	Type 2	30	43	
	Type 3	5	4	
Time from injury to surgery (days)		17.8 ± 28.9	19.2 ± 27.5	0.95

*LIPUS* Low-intensity pulsed ultrasound

* *P* value < 0.05

**Table 2:** Comparison of surgical findings between LIPUS and non-LIPUS groups

	Non-LIPUS treatment group	LIPUS treatment group	*P* value
Type of Acutrak screw (4/5 / Plus)	19/25	24/33	1.00
Screw length (mm)	47.2 ± 4.3	47.7 ± 3.5	0.57
Ratio of the screw length to the fifth metatarsal length (%)	59.7 ± 4.3	60.8 ± 4.4	0.17

*LIPUS* Low-intensity pulsed ultrasound

* *P* value < 0.05

**Table 3:** Comparison of clinical and radiological outcomes between LIPUS and non-LIPUS groups

	Non-LIPUS treatment group	LIPUS treatment group	*P* value
Time to start running (weeks)	6.2 ± 1.4	6.8 ± 2.1	0.21
Time to return to sports (weeks)	13.2 ± 1.8	13.7 ± 3.3	0.65
Time to bone union (weeks)	12.0 ± 3.8	11.9 ± 3.3	0.77
Nonunion (no. of feet)	2	0	0.19

*LIPUS* Low-intensity pulsed ultrasound

* *P* value< 0.05

## Discussion

The most important finding of this study was that LIPUS treatment after intramedullary screw fixation for proximal fifth metatarsal stress fractures did not improve the time to radiographic bone union. In addition, similar clinical outcomes were observed with or without LIPUS treatment.

Proximal fifth metatarsal stress fractures are commonly caused by repetitive loading on the fifth metatarsal [1, 2] and have increased risk for progression to healing delay or nonunion because of the poor blood supply to the proximal metatarsal diaphyseal region of the fifth metatarsal [3]. Additionally, the longer the time to bone union, the higher the risk of nonunion in proximal metatarsal stress fractures (Lawrence and Botte zone 3 fractures) than in Jones fractures (zone 2 fractures) [3, 24]. Furthermore, surgical treatment results in fewer complications such as delayed union, nonunion, and refracture than conservative treatment and is considered the standard treatment for proximal fifth metatarsal stress fractures. In particular, intramedullary screw fixation showed excellent results by compressing the fracture site and interrupting the continuous load and is often performed early after the diagnosis to reduce the time to return to sports [4, 5]. Moreover, proximal fifth metatarsal stress fractures commonly occur in athletes. Many athletes who undergo surgery desire an early return to sports without increasing complications, such as delayed union and nonunion. Return to sports is recommended in metatarsal stress fractures after clear evidence of clinical and radiographic bone union [6, 7]. Early return to sports is a risk factor for the delayed bone union after intramedullary screw fixation in fifth metatarsal stress fractures [11]. In our rehabilitation protocol, return to sports was permitted after the complete radiographic bone union was achieved, as in other studies examining intramedullary screw fixation for proximal fifth metatarsal stress fractures [8–10]. Therefore, the time to radiographic bone union was not influenced by a return to sports in our study.

LIPUS also has a beneficial effect on the repair of stress fractures despite involving different healing processes between stress fractures and complete fractures [20]. There are a few studies on the application of LIPUS to proximal fifth metatarsal stress fractures [11, 22]. Teoh et al. [22] reported that 2 cases with delayed union of proximal fifth metatarsal stress fracture (Lawrence and Botte zone 3 fracture) treated by LIPUS achieved bone union. In terms of the application of LIPUS after surgery, a recent study by Miller et al. [11] examined 37 patients with LIPUS treatment after intramedullary screw fixation for proximal fifth metatarsal stress fracture showed that mean time to bone union was 12.7 weeks and nonunion was only one. However, the definition of the proximal fifth metatarsal stress fracture in this study included Lawrence and Botte zone 2 fractures as well as zone 3 fractures, although excluded Torg type 1 fractures. To the best of our knowledge, there are no current studies in the literature that evaluate the effect of LIPUS treatment after surgery for only proximal fifth metatarsal stress fractures (Lawrence and Botte zone 3 fractures). We hypothesized that LIPUS treatment after intramedullary screw fixation for proximal fifth metatarsal stress fractures would reduce the time to radiographic bone union regardless of the poor blood supply to the proximal metatarsal diaphyseal region of the fifth metatarsal. Since LIPUS induces more new vessel growth in the fracture sites and improves vascular supply through stimulating vascular endothelium growth factor [13], there is a favorable outcome in LIPUS treatment after intramedullary screw fixation for Lawrence and Botte zone 2 and 3 fractures [11]. However, the time to radiographic bone union in the LIPUS treatment group was similar to that of non-LIPUS treatment in this study. In addition, there was no difference found regarding the time to start running and return to sports between the groups. Therefore, routine use of LIPUS cannot be recommended to reduce the time to bone union after intramedullary screw fixation for proximal fifth metatarsal stress fractures.

This study has several limitations. First, the generalizability of our findings is limited because of the small sample size. A post-hoc power analysis revealed the sample size needed to detect a difference of 19,340 cases in each group, which was larger than the current study. Second, this was a retrospective study and had limitations inherent to retrospective studies. Third, the results were limited to radiograph changes and return to sports, and no other objective measures were evaluated. Furthermore, refracture, which is one of the important outcomes of treating proximal fifth metatarsal stress fractures, was not investigated. However, LIPUS is just a bone healing enhancement device and is not considered to affect refracture after bone union. Finally, LIPUS treatment was started immediately after the wound on the fracture site was healed in this study. There is a possibility of a more effective LIPUS treatment regimen for fracture healing when using LIPUS after intramedullary screw fixation after proximal fifth metatarsal stress fractures. In addition, the effect of LIPUS treatment on delayed union and nonunion after intramedullary screw fixation for proximal fifth metatarsal stress was not investigated because LIPUS treatment was started within 3 weeks after surgery in all cases using LIPUS in this study. The rate of complications after intramedullary screw fixation is low, but further studies are needed to confirm the effect of LIPUS treatment on these complications after surgery.

## Conclusions

This study found no evidence of an influence on clinical and radiological outcomes following LIPUS treatment after intramedullary screw fixation for proximal fifth metatarsal stress fractures. Therefore, we cannot recommend the routine use of LIPUS to shorten the time to bone union after intramedullary screw fixation for proximal fifth metatarsal stress fractures.

## Data Availability

The datasets generated and/or analyzed during the current study are not publicly available but are available from the corresponding author on reasonable request.

## Abbreviation

LIPUS: Low-intensity pulsed ultrasound
